# Type I Interferons and Natural Killer Cell Regulation in Cancer

**DOI:** 10.3389/fimmu.2017.00304

**Published:** 2017-03-31

**Authors:** Lena Müller, Petra Aigner, Dagmar Stoiber

**Affiliations:** ^1^Ludwig Boltzmann Institute for Cancer Research, Vienna, Austria; ^2^Institute of Pharmacology, Center for Physiology and Pharmacology, Medical University of Vienna, Vienna, Austria

**Keywords:** type I interferon, interferon signaling, natural killer cells, tumor surveillance, innate immunity, tumor microenvironment, anticancer therapy

## Abstract

Type I interferons (IFNs) are known to mediate antitumor effects against several tumor types and have therefore been commonly used in clinical anticancer treatment. However, how IFN signaling exerts its beneficial effects is only partially understood. The clinically relevant activity of type I IFNs has been mainly attributed to their role in tumor immune surveillance. Different mechanisms have been postulated to explain how type I IFNs stimulate the immune system. On the one hand, they modulate innate immune cell subsets such as natural killer (NK) cells. On the other hand, type I IFNs also influence adaptive immune responses. Here, we review evidence for the impact of type I IFNs on immune surveillance against cancer and highlight the role of NK cells therein.

## Introduction

Type I interferons (IFNs) have been initially identified 60 years ago as antiviral substances (1). They are a family of monomeric cytokines consisting of 14 IFNα subtypes, IFNβ, IFNε, IFNκ, and IFNω. While IFNα and IFNβ have been extensively studied during the past decades, the functions of IFNε, IFNκ, and IFNω remain poorly understood (2, 3). The term type I IFNs in this review therefore refers to the well-characterized forms IFNα and IFNβ, whereas the other type I IFN subtypes have been reviewed elsewhere (4, 5).

**Figure 1:**
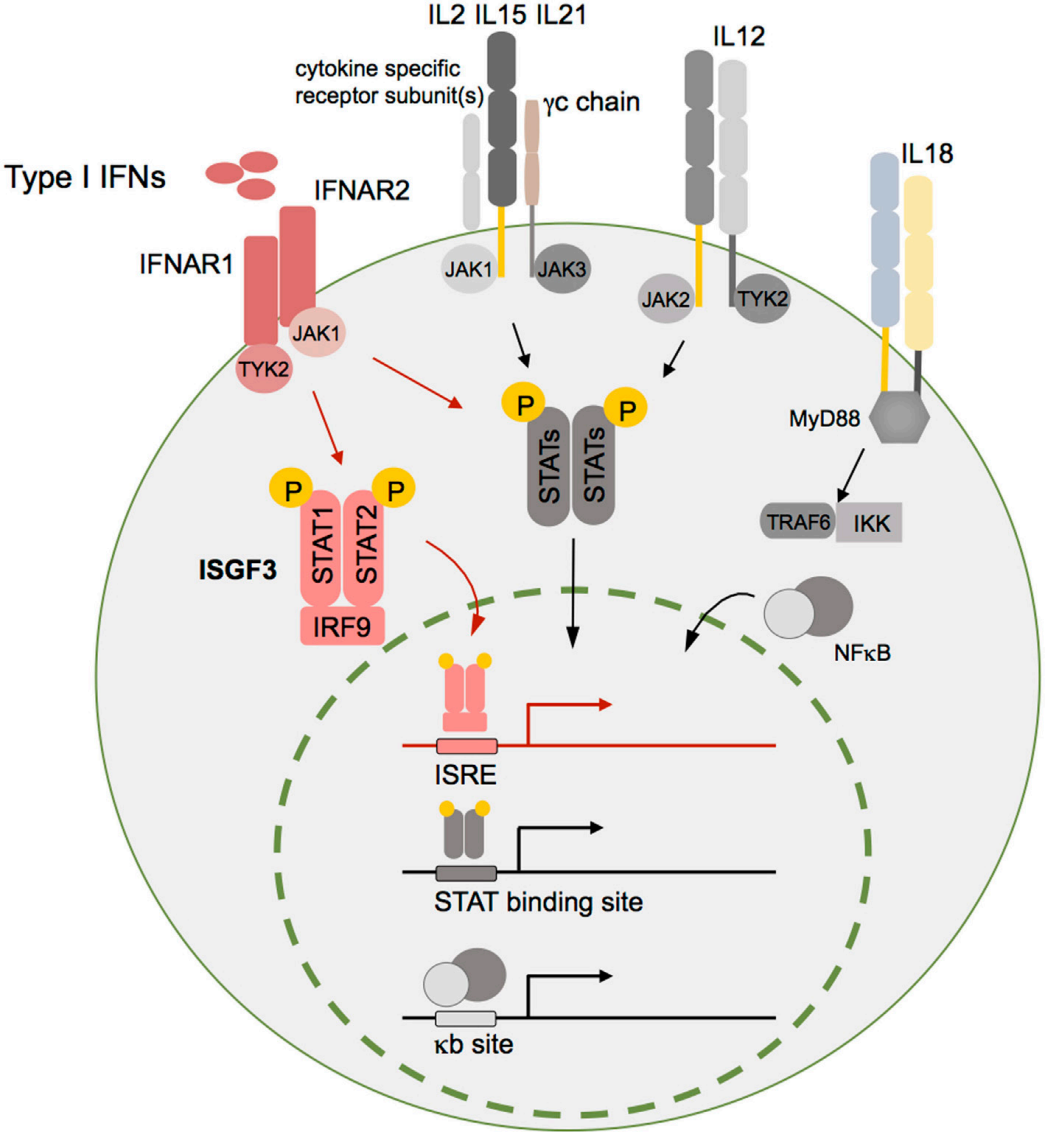
**Type I interferons (IFNs) and different other cytokines are essential for natural killer (NK) cell homeostasis and function**. Although type I IFNs are in focus of this review, additional cytokine pathways such as interleukin (IL)-2, IL12, IL15, IL18, and IL21 are schematically indicated here as important mediators of NK cell function. Cytokine receptor binding triggers downstream signaling pathways such as the Janus kinase (JAK)/signal transducer and activator of transcription (STAT) or nuclear factor kappa B (NFκB) pathway. The respective activated transcription factor complex—IFN-stimulated gene factor 3 (ISGF3) (type I IFNs), STAT dimers (IL2, IL15, IL21, and IL12), and NFκB (IL18)—translocates into the nucleus and induces target gene transcription leading to expression of genes that are crucial for survival, proliferation, differentiation, and cytotoxic function of NK cells. For reasons of simplicity, IL2R, IL15R, and IL21R were summarized in this graph. The receptor-specific subunit(s) in case of IL2R and IL15R refer to the β- and high-affinity α chain and for IL21R only to one specific subunit. Of note, IL15Rα chain is mainly expressed by other cells such as DCs, which is not displayed here. Abbreviations: ISRE, interferon stimulated response element; γc, common gamma chain; MyD88, myeloid differentiation primary response 88; TRAF6, TNF receptor associated factor 6; IKK, I kappa B kinase; NFκB, nuclear factor kappa B.

Type I IFNs can be secreted by most cell types in the body in response to activation of host pattern recognition receptors such as toll-like receptors (TLRs) and retinoic acid inducible gene-I-like RNA helicases that are triggered by bacterial or viral components (6–8). IFNα and IFNβ signal through the interferon α/β receptor (IFNAR), a heterodimeric transmembrane receptor that is composed of the two subunits IFNAR1 and IFNAR2. Following receptor binding, downstream signals lead to the phosphorylation and translocation of signal transducer and activator of transcription (STAT) factors to the nucleus to drive the expression of IFN-regulated genes (IRGs). For type I IFNs, the main STAT signaling complex is formed by IFN-stimulated gene factor 3 consisting of STAT1, STAT2, and IFN regulatory factor (IRF)-9 (3, 9, 10) (Figure 1), however, alternative pathways of IRG stimulation have been described as well (11–13).

It has become well-accepted that functions of IFNα and β reach far beyond antiviral and microbial defense and include the regulation of physiological processes such as cell survival (12), immune cell homeostasis and functions (14), cell cycle, and differentiation (15–17). Many years back, it came as a surprise that constitutively released endogenous IFNα and IFNβ contribute to tissue homeostasis and inhibit malignant cellular transformation (14, 18, 19). Consequently, the finding that type I IFNs have antineoplastic functions stimulated the clinical development of type I IFN anticancer therapies for certain neoplasias.

However, unraveling molecular key mechanisms underlying the antitumor function of type I IFNs remained very challenging for a long time. Recent advances in the development of genetically engineered mouse models have provided useful tools for investigating these mechanisms and continuously improved our understanding of how IFN signaling interferes with tumor development.

### Type I IFNs in Tumor Development

Type I IFNs have been shown to prevent cellular transformation in premalignant cells *in vitro* by sustaining the expression of the tumor suppressor gene p53 (20). Moreover, cell-intrinsic roles for type I IFN signaling in negatively regulating tumor cell proliferation and in triggering apoptosis in different human cancer cell lines have been suggested as well (21). *In vitro* generated findings on direct antineoplastic effects of type I IFNs were substantiated by more recently performed *in vivo* studies, where tissue-specific deletion of IFNAR1 from intestinal epithelial cells increased tumor formation in mice treated with dextran sodium sulfate and the carcinogen azoxymethane to induce colitis (22).

However, a growing number of studies during the past decades provided solid evidence that type I IFNs execute antitumor functions mainly indirectly via stimulating immune cells to rapidly eliminate malignant cells. Owing to the ubiquitous IFNAR expression, type I IFNs have been shown to have crucial regulatory effects on immune cells in the context of inflammatory and viral diseases (2, 23). Thus, cellular mediators of the innate as well as the adaptive immune response may be regulated by type I IFNs in the protection of the host against malignant diseases. Indeed, an increasing number of studies performed during the past decades have supported the idea of an anticancer immune response analogous to the reaction of the host against pathogens.

A study performed by Dunn and colleagues elegantly demonstrated for the first time an essential role of endogenously produced type I IFNs in a process widely known as tumor immune surveillance (24). Unexpectedly and in contrast to IFNγ, type I IFNs were found in bone marrow transfer experiments to act on host hematopoietic cells and not on the tumor cell itself during the formation of a protective antitumor immune response.

The knowledge on how type I IFNs impact on cells of the innate and adaptive immune system in the context of tumor surveillance has been refined in numerous subsequent studies [reviewed in Ref. (21, 25)]. Some of the earliest studies identified an essential role of type I IFNs, particularly, for the function of host antigen presenting cells (26–28). Early produced type I IFNs act on the level of CD8α^+^ dendritic cells (DCs) that are required for the successful activation of tumor antigen-specific cytotoxic CD8^+^ T lymphocytes (CTLs). Based on *in vitro* data, it was demonstrated that type I IFN signaling specifically enhances the ability of CD8α^+^ DCs to cross-present antigens (27), most likely by promoting survival of DCs and enhancing antigen persistence on the cell surface during cross-presentation (21, 29, 30). Moreover, type I IFNs have been shown to promote DC maturation, differentiation, and migration (28).

Finally, type I IFNs induce the release of interleukin 15 (IL15) by DCs (31), thus promoting the survival of CD8^+^ memory cells and NK cells (32), which will be discussed in more detail later on. In response to type I IFNs, CTLs have also been shown to acquire full effector functions (26, 33). Also by impacting on other innate immune cell subsets such as neutrophils (34–38), NKT, and γδ T cells (39), type I IFNs exhibit tumor-growth limiting properties.

In addition, type I IFNs promote a protective antitumor response by inhibiting cells of the tolerogenic tumor microenvironment such as myeloid-derived suppressor cells (MDSCs) (40, 41) and regulatory T cells (Tregs) (42–45) that might interfere with the host tumor immune response.

Type I IFNs are released very early during infections (46), thus it was not surprising that they are important regulators specifically of innate immune cell subsets such as DCs and NK cells in anticancer host responses. For NK cells, type I IFNs have already been demonstrated in viral infection to be critical for early responses and are thought to enhance NK cell cytotoxicity and cytokine production (47, 48). However, how type I IFNs regulate NK cell function in the context of tumor development will be outlined in detail in the following sections.

## NK Cells and Type I IFNs

The importance of NK cells in tumor immune surveillance was initially demonstrated via depletion of NK cells from mice rendering them more susceptible to transplanted tumor cells or methylcholanthrene (MCA)-induced sarcomas (49, 50). Furthermore, NK cells have been shown to control the development of B cell lymphomas that arise in mice deficient for perforin, and NK cells were able to recognize and eliminate some of the tumors in the absence of major histocompatibility complex class I (MHC I) (49, 51, 52). Importantly, impaired type I IFN signaling in NK cells leads to a substantial loss of mature NK cell functions that are essential for efficient tumor cell killing. Initially, the effect of type I IFNs on NK cell homeostasis and development has been studied in mice deficient for IFNAR1 or IFNAR2. In the spleens of those mice, NK cell proportions were significantly decreased and mature NK cells of both genotypes expressed lower levels of the surface molecules CD122, CD11b, and Ly49 C + I (53). Thus, IFNAR-deficient NK cells are reduced in numbers and exhibit impaired cytotoxic capacity (24, 53). The cellular and molecular mechanisms of how type I IFN signaling impacts on NK cells and their effector functions are discussed in detail in this and the following section.

### NK Cell Development and Type I IFN Signaling

Murine NK cells develop in the bone marrow and at alternative sites such as thymus and liver (54–57). However, the majority of NK cells detected in the periphery is likely to have developed in the bone marrow. There, common lymphoid progenitor cells lose their potential to develop into precursor cells of other lineages and differentiate toward an NK cell-restricted precursor cell (NKP) via intermediate stages (58–60). Based on the expression of cell-specific markers and the acquisition of functional competence, NK cell differentiation is subdivided into distinct developmental stages. Natural killer cell-restricted precursor cells express CD122 that enables the cell to respond to IL15, which is the hallmark cytokine of NK cell lineage specification. Natural killer cell-restricted precursor cells progress to a transitory immature NK cell (iNK) stage that is characterized by the upregulation of the pan-NK cell marker NK1.1. The terminal maturation step from iNK cells to mature NK cells (mNK) involves the upregulation of Ly49 receptor family members together with CD11b and DX5. Following their complete maturation, mNK cells egress from the bone marrow and reside in the blood, spleen, liver, lung, and various other organs, where they continue to mature to tissue-specific and functionally distinct NK cell subsets (54). In the periphery, classical stages of NK cell maturation are described based on the expression of CD11b and killer cell lectin-like receptor subfamily G, member 1 as well as loss of CD27 and TNF-related apoptosis inducing ligand (TRAIL) expression (61–64).

We have previously identified an unexpected role for type I IFNs in NK cell development. In IFNAR-deficient mice, type I IFN signaling was dispensable for NK cell maturation in the bone marrow, but lack of IFNAR1 expression on NK cells significantly abrogated peripheral maturation in the spleen. Of note, late stage deletion of *Ifnar1* in mature NK cells (*Ifnar1*^f/f^ Ncr1-iCre mice) did not interfere with splenic NK cell maturation indicating that type I IFNs are required at an earlier stage or by other cells for full NK cell maturation in the spleen (65). The impact on NK cell maturation by systemic type I IFNs was also evidenced by Guan and colleagues (66). By generating mixed bone marrow chimeric mice from *Ifnar^−/−^* and wild-type animals, they showed an intrinsic effect of IFNAR signaling on early NK cell maturation in the bone marrow and also in the liver. In line with results from our study (65), mature NK cell numbers remained unchanged in spleen and blood.

### Memory NK Cells and Type I IFNs

Similar to T cells, NK cells as part of the innate immune system are also able to form an immunological memory and terminally differentiate into memory NK cells. Different educational routes have been described that lead to the formation of NK cell memory by antigen-dependent (hapten- and virus-induced) or antigen-independent (cytokine-induced) mechanisms (67, 68).

Sensitization of mice with haptens in the presence of the pro-inflammatory cytokines IL12, IFNγ, and IFNα leads to hapten-specific memory NK cells in the liver (67, 68). Type I IFNs play an important role herein as hepatic NK cells in hapten-sensitized *Ifnar1^−/−^* (and *Il12^−/−^, Ifng^−/−^*) mice failed to induce contact hypersensitivity after adoptive transfer to the challenged host (69).

Interestingly, in a murine cytomegalovirus (MCMV) infection model, type I IFNs have been proposed to play a role in the differentiation of antigen-dependent memory NK cells. Acute MCMV infection stimulates the production of type I IFNs and other pro-inflammatory cytokines (IL12, IL18, IFNγ, IL21) (70, 71). These pro-inflammatory signals drive the expression of the BTB-ZF transcription factor *Zbtb32* (also known as ROG, FAZF, TZFP, PLZP) in antigen-specific NK cells, which is essential for their proliferation and protective function during MCMV infection (72). By using NK cells deficient for IFNAR1 in mixed bone marrow chimeric mice, Madera et al. demonstrated that direct type I IFN signaling in NK cells promotes their optimal activation and function during MCMV infection. However, type I IFNs were shown to be dispensable for the survival of NK cells and NK memory formation (73).

Also in other virus infection models, type I IFNs and NK cells play important roles. In mice, lytic infection in macrophages with gammaherpesvirus was restricted by NK cells independently of type I IFNs, but spreading of virions to the spleen was only possible in the absence of both, type I IFNs and NK cells (74).

Of note, NK cell memory against tumors has not been observed under physiological conditions. Receptors such as NKG2D that are involved in the recognition of tumor cells by NK cells may not be capable of efficiently generating memory. Moreover, it is also conceivable that host-derived factors such as cytokines in addition to specific ligands for activating NK receptors are needed for the generation of memory NK cells against tumors and that these factors are under-represented in the tumor microenvironment (68). Still, memory NK cells bear the potential to be further manipulated to target tumor cells (see section “Type I IFNs and Anticancer Therapies—A Role for NK Cells Therein?”).

## Interplay of Type I IFNs and NK Cells as Part of the Tumor Immune Surveillance System

### Direct Type I IFN Effects on NK Cell Cytotoxicity

As mentioned above, IFNAR1 as well as IFNAR2-deficient NK cells are diminished in numbers and exhibit considerably reduced cytotoxic capacity. These defects finally translate into severely impaired tumor surveillance in *Ifnar1^−/−^* and *Ifnar2^−/−^* mice, which succumb earlier to carcinogen-induced fibrosarcoma and RMA-S lymphoma (24, 53). These findings were substantiated by the importance of type I IFN signaling on NK cell-mediated *v-Abl* oncogene-driven B cell leukemogenesis (65). In this context, mice with impaired type I IFN signaling (i.e., *Ifnar1^−/−^* and *Ifnb^−/−^* mice) had an increased susceptibility to *v-Abl-*induced leukemia/lymphoma and B16F10 melanoma. Increased tumor incidence in these models is linked to defects in NK cell-mediated tumor surveillance, which is dependent on their reduced cytotoxic capacity. Indeed, NK cells derived from *Ifnar1^−/−^* and *Ifnb^−/−^* animals display impaired cytotoxic effector function against their target cells *in vitro* (24, 53, 65).

In line with reduced cytotoxicity observed in NK cells lacking either IFNAR1 or IFNβ expression, a similar effect has been reported for NK cells deficient for downstream components of the type I IFN pathway, such as TYK2 (75) or STAT1 (47, 76).

Similar to NK cells derived from IFNAR1-deficient mice, NK cells isolated from mice lacking type I IFN signaling only at the mature NK cell stage (*Ifnar1*^f/f^ Ncr1-iCre mice) (65, 77) display a substantial defect in cytolytic capacity against hematopoietic tumor cell lines (YAC-1, RMA-S) *in vitro*. However, challenging these *Ifnar1*^f/f^ Ncr1-iCre mice with the *v-Abl* oncogene revealed that IFN signaling in mature NK cells is dispensable for the surveillance of leukemia (65). This result might be explainable by the complex cytokine milieu *in vivo* compensating for the obvious defects under IL2-dependent *in vitro* culturing. Previous studies showed that *Ifnar1* deficiency severely curtails NK cell cytotoxicity even in the presence of high doses of IL2 (53). Interestingly, exogenous IL12 stimulation significantly enhances the cytotoxicity of *Ifnar1^−/−^* and *Stat1^−/−^* NK cells. Moreover, IL15 stimulation completely restores cytotoxic activity of *Stat1^−/−^* NK cells *in vitro* (47). These findings clearly show that NK cell defects in *Ifnar1^−/−^* or *Stat1^−/−^* animals cannot be overcome by IL2 stimulation, but might be partially compensated by other cytokines *in vivo*. This underscores the importance of other cytokines in NK cell biology such as IL15 and IL21 that are known to increase the cytolytic activity of NK cells *in vivo* (52, 78, 79) (Figure 1). An additional possible explanation is that in *Ifnar1^−/−^* mice other cell types that do require type I IFNs are critically involved in tumor surveillance.

### Indirect Type I IFN Effects on NK Cells via Other Immune Cells

Natural killer cells do not possess immediate and permanent effector functions. A process called “priming” is required to induce the establishment of the entire NK cell competence (80, 81). Natural killer cell priming is dominated by type I IFNs, which provide essential signals for DCs to produce IL15, the master cytokine for promoting NK cell development, proliferation, and function (54, 80, 82–84).

The activation of NK cells can be induced by DCs through pathways that require cell–cell contact (NKG2D-MICA and/or MICB) and cytokines such as IFNα, IFNβ, IL2, IL12, IL15, and IL18 (82) (Figure 1). Dendritic cell-derived signals elicit both NK-cell-mediated cytolysis as well as cytokine production. Resting and activated DCs are capable of activating NK cells, however, the latter far more potently. The interaction between activated DCs and NK cells has been shown to augment the efficiency of NK cell antitumor effector function in different *in vitro* and *in vivo* models (85, 86). Upon type I IFN signal recognition, DCs produce IL15 and trans-present IL15 to resting NK cells (80). Thus, the interaction with DCs equips NK cells for full effector function. In turn, NK cells are also capable of affecting DC functions through their involvement in DC maturation and DC elimination (82).

More recently, myeloid cells came again into focus as a mechanism was proposed on how cells such as DCs and macrophages could assist NK cell-mediated tumor control (87). Dectin-1 expressed on myeloid cells is critical for NK cell-mediated killing of tumor cells that express high levels of N-glycan structures. Receptor recognition of such tumor cells led to activation of IRF5, an IRF best known for its function in pathogen-induced immunity via activation of MyD88-dependent TLR pathway. This Dectin-1-IRF5 pathway activation in myeloid cells led to activation and efficient tumoricidal function of NK cells. The interaction of myeloid cells and NK cells here may be partially dependent on the expression of the IRF3-dependent NK activating molecule, a membrane-bound protein known to activate NK cells via its homophilic interaction (88).

Apart from effects elicited by type I IFNs on myeloid cells, the following mechanisms could also affect NK cell-mediated tumor surveillance. Although most of those mechanisms have been identified in the context of viral infections, they might be of significant importance in the tumor setting.

The interaction of NK and T cells is also influenced by type I IFN signaling. Type I IFNs keep NK cells from eliminating antigen-activated CTLs by modulating the expression of NK cell receptor ligands (89, 90). In the context of lymphocytic choriomeningitis virus infection, Crouse et al. demonstrated that direct sensing of type I IFNs by T cells prevents them from NK cell-mediated killing by keeping the expression of NCR1 ligands on the CTLs low (89). With the same viral infection setting, Xu et al. showed that the elimination of virus-activated T cells by NK cells was inhibited by type I IFN-induced expression of selected inhibitory NK cell receptor ligands, i.e., classical and nonclassical MHC molecules (MHC I and Qa-1b) (90). An effect of type I IFN signaling on MHC I expression and therefore antigen presentation was reported already earlier, however, the differences in MHC I expression on IFNAR1-deficient cells appeared to be of minor extent (91–93).

Another NK cell surface molecule, TRAIL, was reported to be critical for NK antitumor function in mice and humans (94–96). For example, murine liver NK cells contribute to natural antimetastatic function against TRAIL sensitive tumor cells and constitutive TRAIL expression on these NK cells is IFNγ dependent (96, 97). During viral infection, type I IFNs were also described to enhance antiviral response by NK cell cytotoxicity through induction of TRAIL on NK cells (98).

Another aspect of tumorigenesis influenced by type I IFN signaling is oncogene-induced senescence. In this context, DNA-damage-induced production of type I IFNs enhances cellular senescence (99). In addition, type I IFNs produced by senescent cells indirectly stimulate NKG2D ligand expression on senescent malignant cells, thus promoting the elimination by NK cells (100). However, IFNα has been shown to downregulate the expression of NKG2D ligand H60 in MCA-induced tumors in 129/Sv mice resulting in reduced effectiveness of NK target recognition and NK-dependent killing (101). This indicates that depending on the tumor model, type I IFNs differentially regulate NKG2DL expression.

Finally, ligands for receptors of immune checkpoints such as those of the programmed cell death protein 1 (PD1) family are induced by type I IFNs (102, 103). This immunoregulatory function of type I IFNs is of great relevance and needs to be taken into consideration for the design of clinical anticancer treatments. Recently, targeting of PD1-ligand (PDL1), which is recognized by its inhibitory receptor PD1 expressed on NK cells and other immune cell subsets gained a lot of attention in oncology and will be discussed in more detail later on.

**Figure 2:**
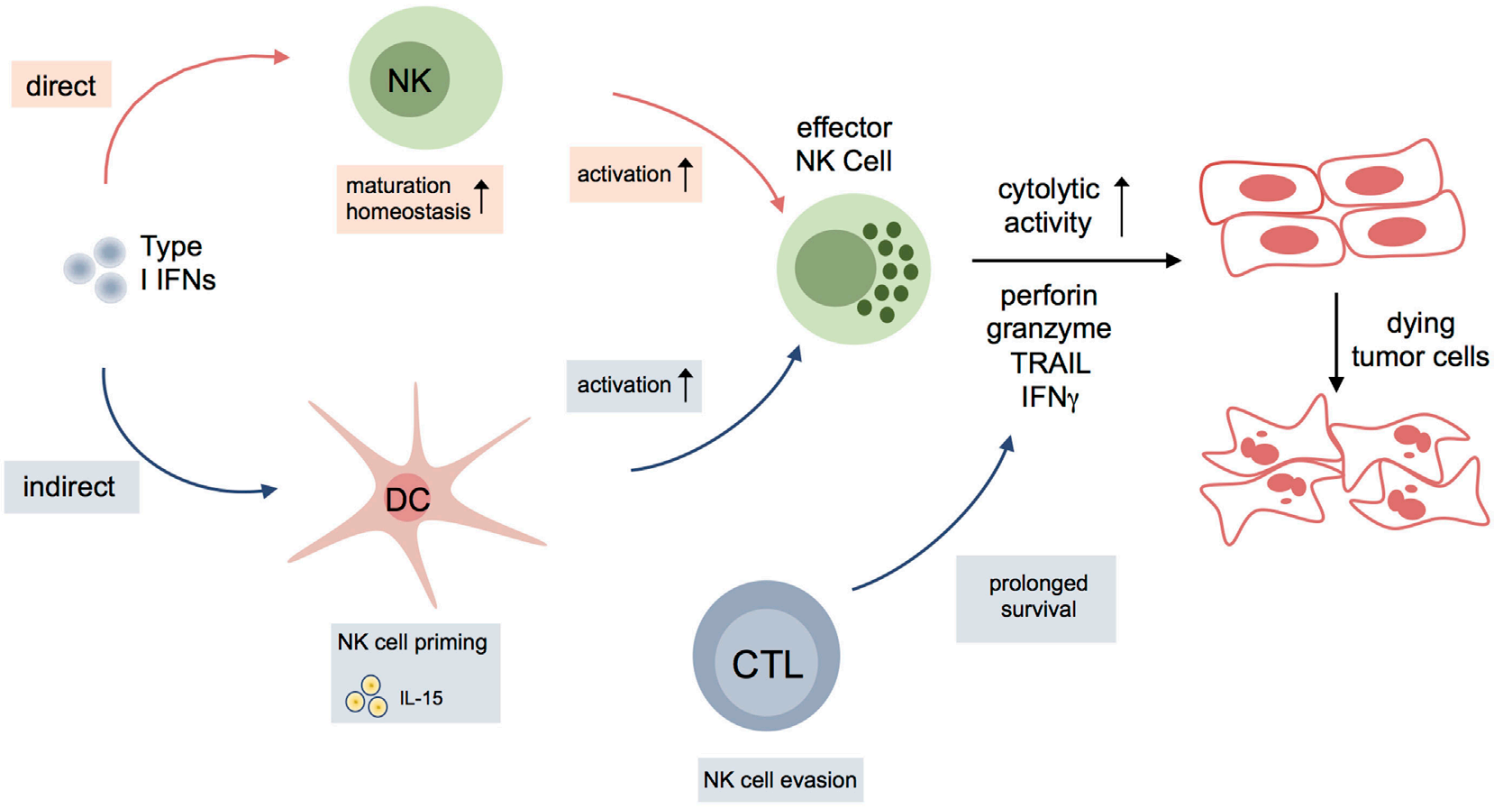
**Interplay of type I interferons (IFNs) and natural killer (NK) cell activation during antitumor response**. Type I IFNs either impact on maturation, homeostasis, and activation of NK cells, or indirectly influence NK cells to kill tumor cells via other immune cells or cells of the tumor microenvironment. Dendritic cells (DCs), in particular, are essential for NK cell priming via production of IL15. Another indirect effect of type I IFNs on NK cell function in cancer might result from modulation of surface molecules on CD8^+^ cytotoxic T lymphocytes (CTLs) [NCR1 ligands; classical and nonclassical major histocompatibility complex class I (MHC I)] leading to evasion of CTLs from NK cell-mediated elimination.

The mechanisms involving NK cells and type I IFN signaling in tumor surveillance are summarized in Figure 2.

## Type I IFNs, NK Cells, and Metastasis

Metastasis as the dreadful consequence of tumorigenesis has recently been shown to be controlled by antitumor immune responses. In this context, NK and CD8^+^ T cells as the main cellular mediators of tumor immune surveillance have been described to be capable of restricting metastatic tumor growth. Therefore, depletion of CD8^+^ T cells or NK cells increased metastasis formation in a breast cancer mouse model without affecting primary tumor growth (104). One mechanism proposed for the metastasis surveillance function of NK cells relies on the inhibition of the MERTK (also known as TAM; TYR3, AXL, and MER) family tyrosine kinase receptors that suppress NK cell activation (105, 106). Of note, the protective function of NK cells against metastases can be also linked to and is partially dependent on type I IFN signaling. In a syngeneic mouse model of mammary tumor metastasis using 4T1.2 cells, Bidwell and coworkers identified a number of IRF7 target genes that are suppressed in bone metastases (104). Consequently, metastasis formation in spontaneous (MMTV-PyMT) and orthotopic mammary tumorigenesis models was accelerated in mice deficient for IFNAR1, NK cell, or CD8^+^ T cell responses (104, 107). Conversely, enforced expression of IRF7 in tumor cells or treatment with type I IFNs enhanced the immune activity and suppressed bone metastasis, thus prolonging survival of the diseased mice. Of note, depletion of both CD8^+^ T and NK cells significantly accelerated metastasis and shortened survival time in mice harboring 4T1.2 tumors ectopically expressing IRF7. This indicates that IRF7-induced and type I IFN-dependent inhibition of bone metastasis was mediated by CD8^+^ T and NK cells (104). In line with the data obtained from metastasis studies in mice, loss of IRF7-associated gene signature in primary tumors of breast cancer patients predicted an increased risk of bone metastasis and also additional studies suggest a suppressive role for type I IFN signaling on breast cancer progression (25).

However, tumor cells use different immune evasive strategies to survive at distinct metastatic sites. The recruitment of immunosuppressive cells is one major mechanism to overcome the immune surveillance system (108). For example, systemic factors from hypoxic breast cancer cells increase myeloid CD11b^+^ cell accumulation and reduce the cytotoxic functions of NK cells in the premetastatic lung (109). Myeloid cells, especially MDSCs, have the capacity to suppress immune responses, thus it is conceivable that recruited myeloid cells establish a premetastatic immune-suppressive niche to promote tumor metastasis. Moreover, platelet activation and the resulting fibrin clot formation support survival of tumor cells that are nested at metastatic sites by protecting them from NK cells (108, 110).

In mice engrafted with mammary tumor cell lines, type I IFN treatment has been shown to reduce metastasis to bone. Interestingly, while MDSC accumulation was substantially decreased, there was an increase in numbers of NK cells present in the bone marrow of these mice (104). Hence, the authors proposed that type I IFNs specifically inhibit bone metastases of mammary cancer by a selective modulation of MDSCs and NK effector cells in the bone marrow (104).

A consecutive study demonstrated that endogenous type I IFN signaling in the host hematopoietic system is indispensable for the responsiveness of circulating NK cells and therefore essential for metastasis-free survival. Consistently, *in vivo* stimulated NK cells derived from *Ifnar1^−/−^* mice but not from wild-type counterparts failed to eliminate the 4T1 and 66cl4 mammary tumor cell lines *in vitro* (107).

In summary, these studies clearly highlight an essential role for IFN signaling and NK cells during metastasis formation and could pave the way for type I IFNs for new therapeutic means in metastatic cancer.

## Type I IFNs and Anticancer Therapies—A Role for NK Cells Therein?

As outlined above, ample evidence substantiates the importance of type I IFN signaling in NK cell-mediated tumor surveillance. Interferons mainly function by modulating the immune system rather than executing direct anticancer effects. In the clinics, type I IFNs have been used for decades as anticancer therapy, however, the exact mechanism of action of type I IFNs has not been clarified yet (111, 112). IFNα has been and is still used mainly for the treatment of hematopoietic neoplasms. Especially, before the advent and breakthrough of the BCR-ABL inhibitor imatinib as therapy for chronic myeloid leukemia (CML), IFNα was the treatment of choice for patients not suitable for bone marrow transplantation. Interestingly, in chronic myeloproliferative neoplasms the positive effect of IFNα coincided with a substantially higher frequency of circulating CD56^bright^ NK cells that produced increased levels of IFNγ (113). Recently, IFNα has gained attention for further use as therapeutic option in CML, preferably in combination with imatinib or its next generation inhibitors (114).

Trials with IFN therapies in solid malignancies have met with varied success. However, besides virus-related cancers at least in melanoma as one type of solid tumors, IFNα is clinically used (21). In high-risk melanoma patients, high-dose IFNα treatment leads to an extension of relapse-free survival and is therefore considered a valid therapeutic option. Interestingly, IFN therapy is more effective at targeting disseminated cancer cells and minimal residual disease before they form large proliferative metastases, emphasizing again that promotion of antitumor immunity rather than direct antiproliferative effects is the predominant mechanism of action (25).

Data obtained mainly from tumor studies in mice strongly suggest that the success of conventional chemotherapeutics (such as anthracyclines, cyclophosphamide), targeted anticancer agents, radiotherapy, and immunotherapy depends on type I IFN signaling (21, 115). Under certain circumstances, this mode of action of IFN signaling involves NK cells. For example, some immunogenic chemotherapeutics lead to the activation of TLR3 in malignant cells by cancer-cell derived RNA which results in type I IFN production. Subsequently, IRGs such as CXC-chemokine ligand 10 (CXCL10) are expressed, which in turn are crucial for recruitment in NK cell-mediated tumor control (116, 117).

The concept of tumor immune surveillance has triggered an increasing interest in immunomodulatory treatment strategies. However, immune-activating therapies are likely to induce the expression of immunosuppressive ligands and receptors such as PDL1, PD1, and CTLA4. Since type I IFNs have immunostimulatory functions, they can promote the upregulation of such surface molecules (102, 118), thus preventing prolonged antitumor immune responses. In this case, a sustained therapeutic antitumor response could be achieved by the combination of type I IFN therapy with other therapeutic means targeting the PD1–PDL1 axis to block secondary immune suppression. Programmed cell death protein 1- and CTLA4-targeted therapeutics have been proven in some cancers to significantly prolong survival of the patients. Combining these agents with type I IFNs could be a suitable strategy to overcome immunosuppression and raise patient responsiveness. Programmed cell death protein 1 is well documented in the context of T-cell responses and has recently been shown to be upregulated on NK cells, which leads to downregulation of anticancer function (110, 119, 120).

On the contrary, IFN signaling seems to be also important for the success of checkpoint immunotherapy, which is illustrated by a recent study on late relapses of PD1 blockade treatment in metastatic melanoma. Here, a loss-of-function mutation in the Janus kinase 1 has been identified in one patient, suggesting that disruption of type I and type II IFN signaling might be involved in preventing the success of checkpoint immunotherapies (121). If this turns out to be a more frequent observation, the combination of type I IFNs with checkpoint inhibitors would be desirable for an improved treatment outcome. Furthermore, in anticancer virotherapy, type I IFNs play a key role, as intratumoral injection of the oncolytic Newcastle disease virus combined with systemic CTLA4 blockade leads to regression of murine B16 melanomas. Interestingly, this effect has been reported to be dependent on CTLs, NK cells, and IFNAR signaling (122).

As outlined above, there are a number of reasons pleading for type I IFNs as tools in anticancer treatment. However, one big disadvantage are dose-limiting side effects, including influenza-like symptoms (fatigue, fever, headache, and muscle aches), nausea, anorexia, dizziness, depression, and leukopenia. To avoid these side effects of IFN therapy, strategies are now being developed to deliver type I IFNs directly to the tumor microenvironment (21). Different types of cells can be manipulated to express type I IFNs to augment their own antitumor activity or to promote the activity of other immune effector cells of the host. This has been also assessed with NK cells: a genetically engineered NK cell line expressing human IFNα displayed improved cytotoxicity functions against hepatocellular carcinoma cells *in vitro*, as well as in xenograft tumor models (123). Moreover, mesenchymal stem cells modified to express mouse IFNα efficiently decreased the growth of murine B16 melanomas *in vivo*, an effect that was shown to be dependent on NK and T cells (90). However, translating this strategy to the clinics might be difficult and other means, such as the usage of modulators of specific immune cell subtypes and/or pathways might be preferred.

As described above, memory NK cells against tumors have not been observed yet, but would be highly appreciable if those could be generated *in vitro* by different manipulations such as transduction of proliferating NK cells with chimeric antigen receptors, or enhanced antibody dependent cellular cytotoxicity (ADCC) using newly identified human FcεRIγ-deficient adaptive NK cells (68). As antigen-dependent memory NK cell formation relies also on type I IFN signaling, this could be another strategy for type I IFNs and NK cells in cancer control.

The studies on type I IFNs and breast cancer metastasis (see section “Type I IFNs, NK cells and metastasis”) may provide a rationale for targeting the endogenous type I IFN pathway as an antimetastatic strategy. As IFN signaling modulates the tumor immune response, targeting type I IFNs to a specific cellular compartment of the tumor mass may mediate optimal therapeutic effects for some cancer types. Type I IFN signaling within tumors is essential for both natural and therapy-induced immune surveillance. Thus, downstream effectors of type I IFN signaling would be suitable candidates for further investigation as prognostic and predictive biomarkers in cancer diagnosis and progression (21).

The high potential and importance of type I IFNs and NK cells in cancer is also illustrated by a glimpse on current clinical trials. Searching for IFNα, NK cells, and cancer at ClinicalTrials.gov resulted in 16 studies, half of them dealing with type I IFNs and NK cells for cancer patients (https://clinicaltrials.gov; November 2016). Already in 1997, Nagler et al. combined type I IFNs with NK cell-stimulating molecules such as IL2 and indeed showed increased survival in lymphoma patients after stem cell transplantation (124). Recently, a more specific approach using adoptive transfer of autologous or allogeneic NK cells is frequently tested for cancer treatment (125). Here, even synergistic or additive effects of type I IFNs applied in this context could be imagined.

The combination of type I IFNs with other immunostimulatory agents such as immune checkpoint blockers, cytokines, or other inhibitors that target different immunosuppressive circuits is likely to result in optimal NK cell anticancer function and tumor control.

## Conclusion and Perspectives

Type I IFNs are essential in antitumor control and execute their function predominantly by modulating the activity of other immune cells. Although type I IFNs affect various immune cell subsets, the impact of type I IFNs on NK cells is especially crucial for efficient tumor immune surveillance. Type I IFNs not only positively regulate NK cell maturation and memory, but also NK cell priming and NK cell-mediated tumor surveillance by various mechanisms. Detailed knowledge about underlying mechanisms of immunoregulatory cell recruitment and their suppressive functions in primary tumors and at metastatic sites should lead to more effective immunotherapies.

Thus, therapeutic approaches will need to include the evaluation of immune cell profiles in individual cancers, so that drug targeting can be precisely tailored to maximize the response. In addition, tumor-type specific treatments of type I IFNs and other therapeutic concepts might extend the pharmacological armament to combat diverse cancer types.

## Author Contributions

LM, PA, and DS have written the review article. LM has compiled graphics for this review article.

## Conflict of Interest Statement

The authors declare that the research was conducted in the absence of any commercial or financial relationships that could be construed as a potential conflict of interest.
